# Microwave-Assisted Synthesis of Nitrogen and Sulphur Doped Graphene Decorated with Antimony Oxide: An Effective Catalyst for Oxygen Reduction Reaction

**DOI:** 10.3390/ma15010010

**Published:** 2021-12-21

**Authors:** Nadia Garino, Adriano Sacco, Angelica Chiodoni, Candido F. Pirri, Micaela Castellino

**Affiliations:** 1 Department of Applied Science and Technology, Politecnico di Torino, Corso Duca degli Abruzzi 24, 10129 Torino, Italy; fabrizio.pirri@polito.it (C.F.P.); micaela.castellino@polito.it (M.C.); 2 Center for Sustainable Future Technologies @Polito, Istituto Italiano di Tecnologia, Via Livorno 60, 10144 Torino, Italy; angelica.chiodoni@iit.it

**Keywords:** doping, reduced graphene oxide, antimony, oxygen reduction reaction, microwave

## Abstract

In this study, we report on the facile synthesis of a novel electrocatalysts for the oxygen reduction reaction (ORR), based on reduced graphene oxide (RGO), functionalized with metallic and non-metallic elements. In particular, thanks to a fast one-pot microwave-assisted procedure, we induced, in the RGO graphene lattice, a combined doping with nitrogen and sulphur, and the simultaneous decoration with antimony oxide nanocrystals. The multi-doped–decorated material shows enhanced catalytic performance towards ORR, with respect to common nitrogen- or sulphur-doped carbon-based materials. The presence of co-doping is confirmed by transmission electron microscopy and X-ray photoelectron spectroscopy analysis. The detailed electrochemical characterization shows the simultaneous effects of dopant atoms on the catalytic behavior. In particular, the importance of nitrogen and sulphur atoms in driving the oxygen absorption, together with the role of antimony in enhancing the electrochemical performance toward the ORR, are discussed.

## 1. Introduction

In the global challenge to reduce the use of fossil fuels and the consequent emissions of greenhouse gases, the development of novel, efficient and sustainable energy conversion devices attracts large interest from the scientific community. In this perspective, metal–air batteries [1] and fuel cells are two of the most promising systems [2] capable of facing the need for alternative solutions. Nevertheless, there are still some critical issues that need to be solved before their large-scale commercialization. In particular, the improvement of such devices is strictly connected with the design of efficient and low-cost electrodic materials, with special attention to the cathodic compartment at which the oxygen reduction reaction (ORR) occurs [3]. 

Limitations in real applications, such as ORR high overpotentials and very slow kinetics, must be overcome by employing a catalyst that is able to drive the reaction with high efficiency towards the four-electron pathway, allowing direct oxygen reduction in a single step. In particular, it is important to hinder the two-electron pathway (which is in competition with the direct four-electron one), because it reduces the system efficiency and produces hydrogen peroxide, which is corrosive and poisonous for cell components [4].

Nowadays, the most widely used ORR catalysts are based on noble metals (in particular Pt) nanoparticles or clusters, supported on carbon nanostructures. Such active materials present high cost [5], low abundance and deteriorative activity of the catalyst, related to noble metal poisoning [6].

For this reason, in the last two decades, many efforts have been spent in studing alternative materials to Pt. In this sense, noble metal-free amorphous carbon or graphene-based electrocatalysts have been widely considered as the most promising alternatives for ORR, thanks to their encouraging performance, the excellent electron transport properties of graphene-like materials, their long-term stability and, not least, their low cost [7]. Especially, carbon lattice doping [8], or its decoration with transition metal oxide nanostructures [9,10], has proved to be a winning strategy, by increasing the electrochemical selectivity toward the ORR four-electron pathways. 

In this work, we report on the easy and fast synthesis of a novel ORR catalyst that combines (i) the carbon lattice doping with nitrogen and sulphur atoms and (ii) its surface functionalization with antimony oxide (Sb_2_O_3_) nanocrystals. The carbon lattice is therefore activated by the presence of nitrogen and sulphur catalytic centers, which affect the carbon electronic structure. This increases the oxygen adsorption and reduces the activation energies requested for the ORR steps [11]. 

In fact, the active role of nitrogen doping in its different possible configurations (pyridinic, graphitic and pyrrolic) [12] is widely known and understood, while the presence of sulphur doping acts in the electron transport mechanism and towards the electrolyte ions migration, thanks to its peculiar electronic structure [13]. On the other hand, Sb-based materials have been reported as catalyst substrates used to improve the stability in polymer electrolyte fuel cells (generally as doping element of tin oxide) [14] and recently they have been also considered as potential active materials for new generation batteries [15,16]. In the present study, it is shown that the presence of antimony oxide nanocrystals further increases the catalytic selectivity, by reaching values close to those of standard Pt-based catalysts [17].

In addition, we propose an innovative and reliable synthetic route: a one-pot microwave-assisted hydrothermal process. Starting from graphene oxide (GO) and thanks to the microwave irradiation, it is possible, in a single step, to obtain the reduction in the GO and the simultaneous doping and functionalization of graphene lattice. In detail, by using thiourea as chemical precursor, it is possible to obtain the doping with both nitrogen and sulphur atoms and, by employing antimony acetate, the decoration of graphene flakes with antimony oxide nanocrystals [18]. The obtained catalysts were named as N-S-RGO/Sb.

The structural characteristic and the morphology of as prepared catalyst were fully characterized by means of X-ray diffraction (XRD) and transmission electron microscopy (TEM). Through X-ray photoelectron spectroscopy (XPS) it was possible to confirm the doping and the complete surface chemistry definition. After the structural characterization, the electrocatalytic tests were carried out using cyclic voltammetry (CV), a rotating ring electrode (RDE), a rotating ring-disk electrode (RRDE) and chronoamperometry (CA) measurements, in order to define the catalytic efficiency towards the ORR. 

## 2. Materials and Methods

### 2.1. Catalyst Synthesis

The N-S-RGO/Sb nanocomposite was prepared by following the synthesis procedure reported below. All the synthesis products were used as purchased without any additional treatments.

In a microwave 100 mL Teflon reactor, provided with pressure and temperature sensors (FlexyWave, Milestone Inc., Shelton, CT, USA), 50 mg of GO (Graphenea Inc., Donostia, Spain) were dissolved in 30 mL DI water, with 20 mg of thiourea (Sigma-Aldrich, Milano, Italy). Then, 21.5 mg of antimony (III) acetate (Sigma-Aldrich, Milano, Italy) were put and dissolved in the aforementioned mixture. Subsequently, the precursor mixture was sonicated for about 30 min and the resulting slurry was microwaved for 15 min at 180 °C (800 W maximum power). The vessel was then chilled to room temperature. The final suspension was then gathered in tiny vessels, washed using DI water and freeze-dried in order to remove all the water.

### 2.2. Structural, Morphological, Compositional and Electrochemical Characterization

XRD was performed in Bragg−Brentano symmetric geometry by using a PANalytical X’Pert Pro instrument (Cu Kα radiation, 40 kV and 30 mA) (Malvern Panalytical Ltd., Malvern, UK) equipped with an X’Celerator detector, in the range 10° ≤ 2θ ≤ 90°. Characteristic crystallographic parameters (interlaminar space (d002); crystal stack height (Lc); in-plane crystallite size (La) and number of graphene layers in the crystal (Nc)) were determined as reported in [19].

Samples for TEM were obtained by dispersing, with the support of an ultrasonic bath, the prepared nanocomposite powder in ethanol. A drop of the dispersion was then collected and put on a commercial holey carbon TEM Cu grid, and allowed to evaporate the solvent. TEM analyses were carried out with a FEI Tecnai F20ST (Thermo Fisher Scientific, Waltham, MA, USA) with a field emission gun (FEG), operating at 200 kV, both in TEM and STEM modes.

A PHI 5000 Versaprobe X-ray photoelectron spectrometer (Physical Electronics, Chanhassen, MN, USA), equipped with a monochromatic Al K-alpha X-ray source (1486.6 eV), was utilised to check the composite surface chemical composition. A circular spot of 100 μm in diameter was selected to gather the photoelectron signal for both the high resolution (HR) and the survey spectra. All samples were subjected to a combined electron and Ar ion gun neutralizer system, to decrease the electrical charging effect during the analysis. The semi-quantitative atomic concentration and fitting procedures were acquired using CasaXPS 2.3.23 dedicated software (Casa Software Ltd., Wilmslow, UK). All core-level peak energies were referenced to C1s peak at 284.5 eV and the background contribution in HR scans was subtracted by means of a Shirley function.

A CHI760D electrochemical workstation (CH Instruments Inc., Austin, TX, USA) and a RRDE-3A rotating ring–disk electrode apparatus (ALS, Tokio, Japan) were employed for the electrochemical characterizations. The catalyst samples were deposited onto a glassy carbon disk/Pt ring working electrode (electrode area 0.1256 cm^2^) following the procedure reported in [20]. Unless otherwise specified, all the measurements were carried out in 3-electrode configuration (disk as working electrode/Ag/AgCl as reference electrode/Pt coil as counter electrode) in oxygen-saturated 0.1 M KOH aqueous electrolytic solution with 2500 RPM rotation speed. All the potentials are always referred to the reversible hydrogen electrode (RHE).

For the CV tests, the potential was scanned from 0.18 V to 1.18 V with a rate of 10 mV/s in both O_2_- and N_2_-saturated electrolytic solutions. For the RDE, the potential range was 0.18 V–1.18 V with a scan rate of 5 mV/s, while rotation speed was varied in the range 400–2500 RPM. For the RRDE measurements, a 4-electrode configuration (disk/ring/reference/counter electrodes) was adopted. The disk electrode was scanned from 0.18 V to 1.18 V (scan rate 5 mV/s) and the ring electrode was fixed at 1.18 V. For the CA, the potential was fixed at 0.68 V. 

The results obtained on N-S-RGO/Sb were compared to those of a commercial available material, namely a Pt/C catalyst (20 wt%, Sigma-Aldrich, Milano, Italy).

## 3. Results and Discussion

### 3.1. Structural and Morphological Analysis

**Figure 1:**
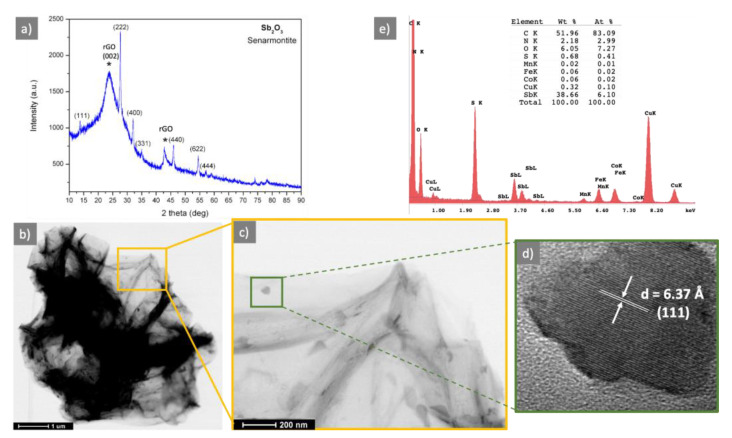
Structural and morphological characterizations of N-S-RGO/Sb sample. (**a**) XRD (* indicate rGO reflections); (**b**) STEM characterization of a N-S-RGO/Sb flake; (**c**) magnification of the orange region in (**b**); (**d**) high resolution TEM characterization of a representative crystal decorating the N-S-RGO/Sb flake; (**e**) EDX spectrum of N-S-RGO/Sb sample.

The structural characteristic of the N-S-RGO/Sb sample was evaluated with X-ray diffraction. In Figure 1a, the XRD pattern of the N-S-RGO/Sb sample is reported. It shows two peak groups. The first, labelled with asterisk, with a main [001] peak at 23.88°, and a second [100] peak located at 42.93°, is assigned to RGO. By analizing the RGO peaks as reported in [19], a d_002_ of 0.37 nm, a crystal stack height Lc of 2.31, an in-plane crystallite size La of 4.95 and number of graphene layers in the crystal Nc of 6.19 have been found. With respect to the well-known graphite d_002_ peak at 0.34 nm, the d_002_ value takes into account both the graphene oxide reduction process and the N and S doping, which, on the whole, contribute to the widening of the RGO peak, thus evidencing a material with an increased structural desorder with respect to grahene or graphite. The d_002_ of 0.37 nm is in line with those found for other graphene oxide reduction techniques [19], while for the other parameters, they are in the same range of other RGO materials or slighly higher (number of graphene layers in the crystals) than those reported. 

The second set of peaks has been assigned to Sb_2_O_3_ (senarmontite JCPDL 01-071-0365). The Sb_2_O_3_ peaks are very sharp, giving evidence of a good crystalline quality. No other crystalline phases, e.g., metallic Sb or other antimony oxides, have been identified. 

The morphological feature of the sample has been assessed wih transmission electron microscopy. In Figure 1b the STEM image shows a representative micrometric flake of N-S-RGO/Sb, composed of several layers. It can be noticed that, after the GO reduction/doping process, the RGO structure is not damaged and the layers—although in some part wrapped—appear as good quality layers. The STEM magnification in Figure 1c shows elongated cystals, about 100 nm long, decorating the single layers of N-S-RGO/Sb flake. These crystals, as evident from the high resolution TEM image in Figure 1d, are single crystals, identified as Sb_2_O_3_ senarmontite, thus confirming the XRD findings. 

Energy dispersive X-ray (EDX) spectroscopy has been used to confirm the presence of N and S in the sample. In Figure 1e the EDX spectrum is reported, together with a semiquantitative elemental analysis. It shows clearly the presence of C, N, S, Sb and O, together with Fe and Co, due to the electromagnetic lenses, Cu, due to the copper grid and a contamination of Mn.

### 3.2. XPS Analysis

**Figure 2:**
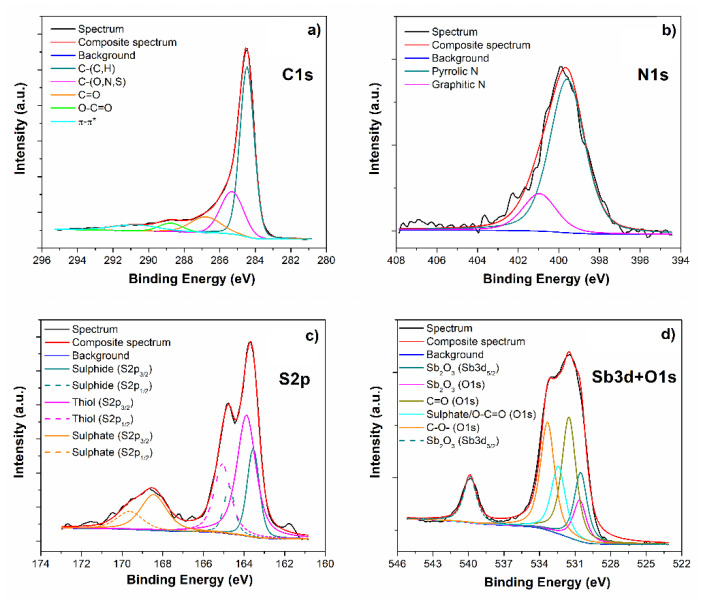
N-S-RGO/Sb XPS HR spectra of C1s (**a**), N1s (**b**), S2p (**c**) and Sb3d+O1s (**d**) regions, together with their deconvolution procedures.

XPS analysis has been performed in order to obtain detailed information regarding element oxidation states, their chemical enviroment and relative allotropic form. Survey spectrum (not reported) highlights the presence of C (82.6 at.%), O (11.5 at.%), N (3.2 at.%), S (2.6 at.%) and Sb (0.2 at.%). No further elements have been detected. The C1s HR spectrum (see Figure 2a) shows a well-reduced graphene oxide signal, since the chemical shifts due to C and O bonds have been highly reduced, if compared with a starting commercial GO material [21]. From the deconvolution procedure, we obtained five pseudo-Voigt curves, which have been associated to: sp^2^ C-(C,H) at 284.5 eV, C-(O,N,S) at 285.3 eV, C=O at 286.8 eV, O-C=O at 288.7 eV and the π−π* transition at 290.7 eV [20]. The N1s peak (Figure 2b) shows the well-known chemical shifts due to nitrogen atoms doping in a graphene-like matrix, due to pyrrolic-N (399.6 eV) and graphitic-N (400.9 eV)—already discussed in our previous work [22]. The second dopant element, introduced in the graphene structure thanks to the use of thiourea, which reduces and dopes the C-based material simultaneously, is sulphur. Its HR spectrum (Figure 2c) shows the presence of multiple peaks, due to the cohesistence of three different oxidation states, due to: sulphide species (163.5 eV), thiol group (163.9 eV) and sulphate (168.5 eV) [23]. We can easily affirm that no M-SbS_2_ (where M is a metal) or Sb_2_S_3_ are present in this material, since the chemical shifts expected for this kind of bonds should be located in the range 161.1–161.6 eV [24,25], while our S2p spectrum does not show any component in that region. In order to obtain information from the Sb3d doublet (Figure 2d), we had to deconvolute its signal starting from Sb3d_3/2_ peak, since the Sb3d_5/2_ overlaps with O1s signal. Hence, the intensity and binding energy of Sb3d_5/2_ peak have been set by the Sb3d_3/2_ peak position and intensity, according to the spin–orbit splitting (Δ = 9.39 eV) and ratio (0.7), and the remaining area is attributed to O 1s signal. As shown in Figure 2d, the Sb3d doublet shows only one oxidation state related to Sb^3+^ [26], in accordance with electron microscopy results previously reported. O1s contribution has been deconvoluted in four peaks associated with Sb_2_O_3_ (530.6 eV), C=O (531.5 eV), sulphate/O-C=O (532.4 eV) and C-O- (533.3 eV) [23]. Thus, we have been able to detect only the presence of antimony oxide (Sb^3+^), which is in accordance with the hypothesis of Sb_2_O_3_ nanoparticles decoration of the graphene matrix, and no doping by Sb can be supported. Moreover, both the absence of a component in the region 529.1–529.6 eV and a peak at 396.7 eV (in the N1s region, see Figure 2b), which is commonly attributed to SbN bond [27], let us confirm also the absence of a direct bond between Sb and N.

### 3.3. Electrochemical Characterization

**Figure 3:**
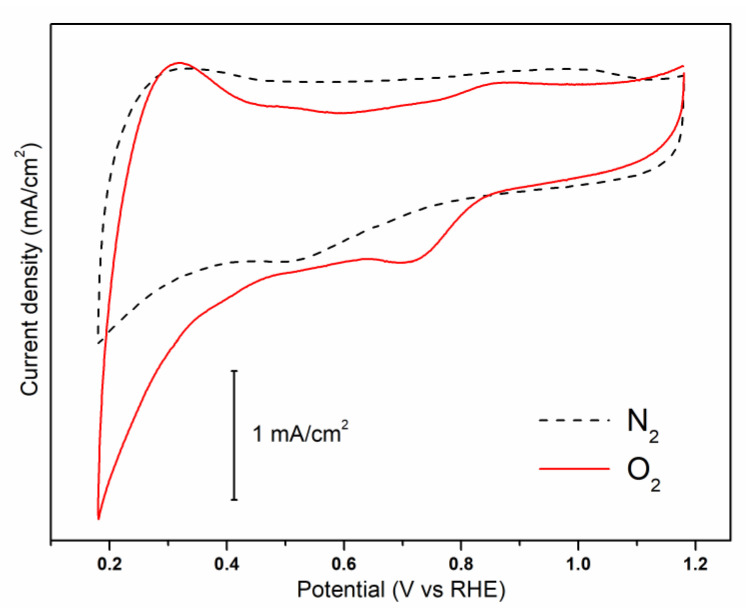
Cyclic voltammograms of the N-S-RGO/Sb catalyst in O_2_- and N_2_-saturated solutions.

The capability of N-S-RGO/Sb composite of carrying out the ORR was assessed through CV measurements. As shown in Figure 3, a reduction peak is present at about 0.72 V when the curve is acquired on oxygen-saturated solution, while it disappears in nitrogen-saturated electrolyte. This peak can be associated with oxygen reduction, in agreement with previously reported results on different graphene-based electrocatalysts [28,29,30]. Moreover, it is important to notice that the sample exhibits supercapacitor characteristics, as witnessed by the quasi-rectangular shape of the CV. This is typical of high surface area carbon-based materials [31], and suggests the possible employment of the N-S-RGO/Sb composite as an active material for supercapacitor applications [32].

**Figure 4:**
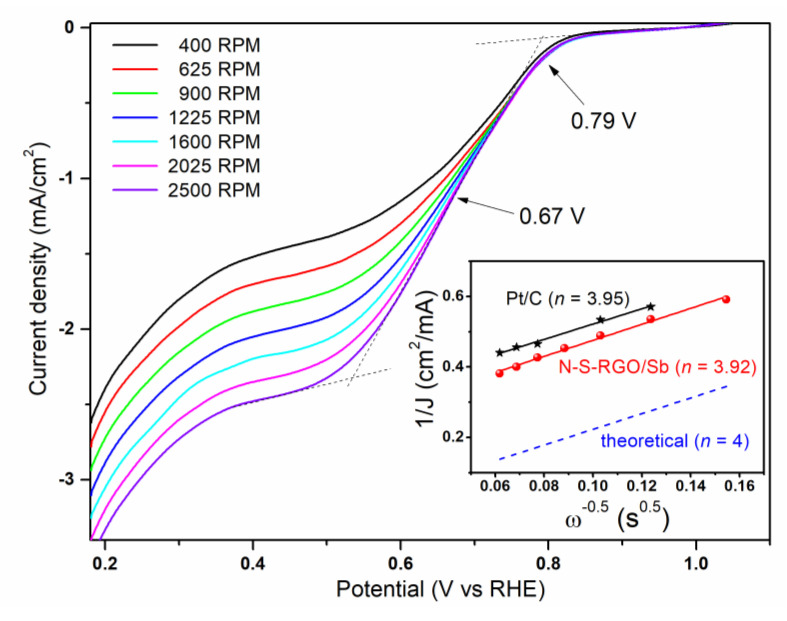
ORR polarization curves of the N-S-RGO/Sb catalyst at different rotation speeds (the arrows indicate the onset and half-wave potentials). The inset reports the Koutecky–Levich plots of N-S-RGO/Sb and of reference Pt/C at 0.38 V potential (the numbers between parentheses represent the calculated electron transfer number values).

In order to further characterize the ORR activity of N-S-RGO/Sb, RDE measurements were carried out at different rotation rates, as reported in Figure 4. While moving from more positive toward less positive potentials, the curves are characterized by a sudden increase in the cathodic current, *J*, at about 0.79 V. This onset potential (*E*_onset_) value points to an activation overpotential of 440 mV for the four-electron ORR, which is slightly higher than 290 mV for reference Pt/C catalysts [33]. The curves then exhibit a quasi-plateau region for potential *E* lower than 0.5 V. In this region, the diffusion-limiting current values become larger while increasing the rotation speed, *ω*, since the diffusion distance reduces at higher rotation speeds [34]. Finally, for *E* ≤ 0.35 V, *J* starts to increase again due to the hydrogen evolution reaction [35]. By considering the curve acquired at 2500 RPM, the calculated half-wave potential *E*_1/2_ (i.e., the potential at which the current density is equal to the half of the diffusion-limiting value) is 0.67 V, a value which is similar to [36] or even better [37] than other graphene-based electrocatalysts reported in the literature. By fixing the applied potential, and reporting 1/*J* as a function of 1/√*ω*, it is possible to obtain the Koutecky–Levich (K–L) plots, which can give information on the selectivity toward the ORR products [38]. In particular, the number, *n*, of electrons involved in the reaction can be obtained by the slope of the K–L plots exploiting the following formula:

(1) $$\frac{1}{J}=\frac{1}{0.62nFC_{O_{2}}D_{O_{2}}^{2/3}\upsilon^{-1/6}\omega^{1/2}}+\frac{1}{J_{K}}$$

where *J*_K_ is the kinetic current density, *F* is the Faraday constant (equal to 96,485.3 sA/mol), *C*_O__2_ is the oxygen bulk concentration (1.2 × 10^−6^ mol/cm^3^), *D*_O__2_ is the oxygen diffusion coefficient (1.9 × 10^−5^ cm^2^/s) and *υ* is the kinematic viscosity of the electrolyte (9 × 10^−3^ cm^2^/s). The result of the calculation for *E* = 0.38 V is reported in the inset of Figure 4. The N-S-RGO/Sb composite is characterized by *n* = 3.92, which witnesses a predominant direct reduction to hydroxide ions, rather than indirect reduction to peroxide [39]. It is worth noting that such a high value is among the largest ever reported for graphene-based materials [40,41] and quite close to that of the reference Pt/C (3.95) and to the theoretical one (i.e., 4), as shown in the inset of Figure 4. This conclusion assesses the intriguing properties of the proposed catalyst.

**Figure 5:**
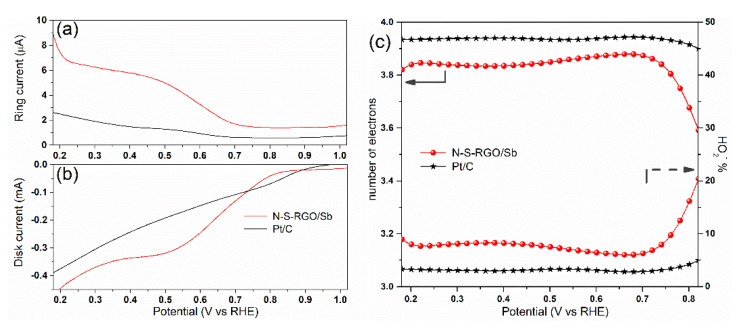
(**a**) Ring and (**b**) disk currents of N-S-RGO/Sb and of reference Pt/C measured at 2500 RPM rotation speed; (**c**) comparison of electron transfer number (left axis) and peroxide percentage (right axis) calculated from the curves in (**a**,**b**).

The outcomes of the K–L analysis were successfully confirmed by the RRDE measurements, whose results are shown in Figure 5. In particular, the ring current *I*_R_, which is associated with the production of peroxide species [42], results two orders-of-magnitude lower, with respect to the corresponding disk current *I*_D_ for *E* ≤ 0.7 V. This implies a predominant 4-electron pathway for N-S-RGO/Sb, similarly to commercial Pt/C [43]. The percentage of produced peroxide species *HO*_2_*^−^%* and the number of electrons as a function of the potential can be calculated by employing the measured *I*_R_ and *I*_R_ through Equations (2) and (3), as follows:

(2) $${HO_{2}}^{-}\%=200\times \frac{I_{R}/N}{I_{D}+I_{R}/N}$$

(3) $$n=4\times \frac{I_{D}}{I_{D}+I_{R}/N}$$

where *N* is the current collection efficiency of the Pt ring. Selectivities toward hydroxide larger than 90% can be obtained with N-S-RGO/Sb for *E* ≤ 0.7 V, with corresponding *n* values larger than 3.8 in this potential range (Figure 5c). The obtained results are only slightly lower than reference Pt/C catalysts, i.e., *HO*_2_*^−^%* ≤ 5%, *n* ≥ 3.9.

It is important at this point to clarify the role of the co-doping and the antimony oxide decoration on the ORR performance. Based on our previous study, in which we fabricated N-doped RGO employing urea as reducing agent instead of thiourea [22], we can affirm that the co-doping is effective in improving the selectivity toward the direct reduction. In fact, the bare nitrogen doping guarantees a mixed 2–4-electron pathway (*n* = 3.38), while this novel co-doped catalyst is characterized by predominant 4-electron reduction. Moreover, we already demonstrated [9,22] that the decoration of N-RGO with transition metal oxides (Sn, Fe) further enhances the ORR selectivity. We thus expect that the same role can be played here by the Sb_2_O_3_ nanocrystals.

**Figure 6:**
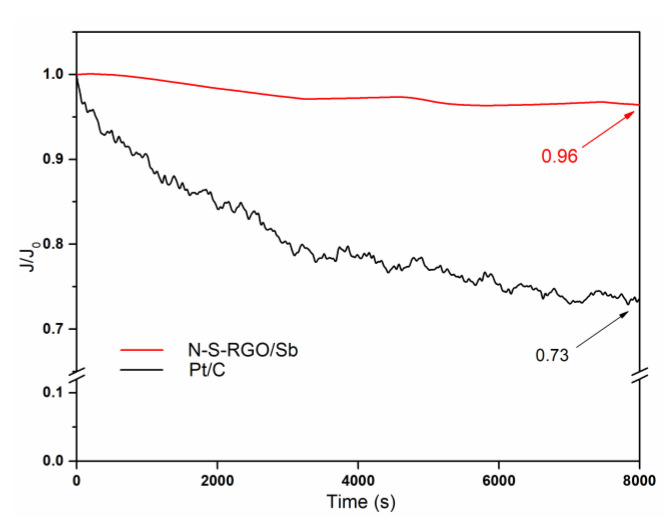
CA of N-S-RGO/Sb and of reference Pt/C measured at 0.68 V potential and 2500 RPM rotation speed normalized with respect to the initial current value (the arrows indicate the values of the normalized currents after 8000 s).

In addition to the promising properties reported above, the N-S-RGO/Sb catalyst is also characterized by enhanced durability with respect to the commercial Pt/C. In fact, due to different reasons, such as the detachment of Pt nanoparticles from the C matrix or upon their aggregation, the ORR activity of the reference catalyst is subjected to a decrease over time [44]. As reported in Figure 6, after 8000 s, the current related to this catalyst is equal to the 73% of the initial value. On the contrary, the novel N-S-RGO/Sb composite exhibits a decrease of just 4% in the same period of time. This result can be attributed to the low production of peroxide species in non-precious metal-based catalysts during the reaction [45]. Moreover, we hypothesize that the presence of antimony is beneficial to futher improve the long term stability of the novel catalyst, in agreement to what was reported in [14].

## 4. Conclusions

In this work, we propose the facile microwave-assisted synthesis of a promising ORR catalyst based on a RGO nanocomposite, presenting the graphitic surface functionalization with Sb_2_O_3_ nanocrystals and the co-doping with both nitrogen and sulphur atoms. The synthesis process was evaluated as possible strategy for the preparation of novel graphene-based materials capable of producing, in a single step, doped and/or decorated nanocomposites without damaging the graphitic lattice and removing the need of additional thermal treatments. The results confirm that microwave-assisted processes can be considered as possible substitutes for traditional synthesis procedures, being cost-effective, by drastically reducing time and energy consumption and at the same time enhancing the homogeneity of the final material. 

The structural and morphological characterizations of the as-prepared catalyst confirmed both the doping and decoration with nanoparticles. In particular, this N-S-RGO/Sb nanocomposite exhibits very good performance toward ORR, thanks to the electrochemical cooperation between the co-doping and the Sb_2_O_3_ nanocrystals. In fact, this specific configuration showed a synergic effect towards ORR, since the presence of nitrogen and sulphur atoms, as carbon lattice dopants, improved the oxygen absorption and the charge transfer properties; moreover, the presence of antimony oxide nanoparticles further enhanced the efficiency and stability of the catalytic process towards the four electrons pathway.

These findings indicate that this peculiar catalyst can be a potential low-cost and highly durable electrocatalyst for fuel cells and metal–air batteries application, by showing an excellent behavior for the ORR, with electrochemical performance consistent with that of commercial reference Pt/C catalyst.

## Data Availability

The data presented in this study is available on request from the corresponding authors.
